# Impact of COVID-19 on Micronutrient Adequacy and Dietary Diversity among Women of Reproductive Age from Selected Households in Bangladesh

**DOI:** 10.3390/nu15143202

**Published:** 2023-07-19

**Authors:** Tasmia Tasnim, Kazi Muhammad Rezaul Karim

**Affiliations:** 1 Department of Nutrition and Food Engineering, Daffodil International University, Daffodil Smart City, Birulia, Savar, Dhaka 1216, Bangladesh; tasmia.nfe@diu.edu.bd; 2 Institute of Nutrition and Food Science, University of Dhaka, Dhaka 1000, Bangladesh

**Keywords:** women of reproductive age, dietary diversity, nutrient adequacy, COVID-19, Bangladesh

## Abstract

Women of reproductive age (WRA) are recognized as a nutritionally sensitive demographic that is vulnerable to micronutrient deficiencies. The purpose of this study is to determine the situation and influencing factors of diet diversity and micronutrient adequacy during the pandemic-induced economic lockdown period among women living in a selected area of Bangladesh. Twenty-four-hour dietary recall was used to measure the nutrient intake and also used for constructing the Minimum Dietary Diversity for Women (MDD-W) and nutrient adequacy ratio (NAR). Household food insecurity and coping strategies were also measured. Multivariate logistic regression was carried out to identify the link between potential risk factors and MDD-W. About two-thirds (59.9%) of the study subjects did not meet the MDD-W threshold. The women’s total energy and protein consumptions were 1475.1 kcal and 46.3 g, respectively, with the diversified diet group consuming more than the non-diverse diet group. Except for vitamin C, vitamin A, and vitamin D, all micronutrients evaluated in the diversified diet group had significantly higher NAR values than the non-diverse diet group. The mean adequacy ratio (MAR) of the overall reproductive women was 0.468 ± 0.096, and it was significantly associated with MDD-W. Another notable finding is that attainment of minimal diversity was not sufficient to achieve acceptable nutrient adequacy for women, pertaining to their low-quantity intake. In addition to this, household size, women’s education, coping strategy, and the MAR were found to be significant determinants of MDD-W in the multivariate logistic regression analysis. The findings of the present study therefore highlight the impending need for interventions that ensure good dietary quality for women even during crisis periods.

## 1. Introduction

Women of reproductive age (WRA) are recognized as a nutritionally sensitive demographic. This is due to their greater physiological demands, which are mostly connected to their capabilities of reproduction, including an increased need for nutrition during menstruation, pregnancy, and breastfeeding [1]. Women’s nutritional needs require special attention, particularly during their developing and maturing stages, because nutrient deficiency during this critical time will impact women’s current and future well-being by increasing their vulnerability to illnesses and impairing their physiological growth, mental development, and efficiency of work [2]. Aside from pregnancy and breastfeeding, women need a more nutrient-rich diet than men because they are smaller and eat fewer calories [3]. Micronutrient deficiencies are common among WRA in developing countries [4], and they are exacerbated during pregnancy. These inadequacies, if not addressed properly, can result in poor perinatal outcomes and an increased risk of maternal and neonatal death.

Researchers who have studied women’s diets in LMICs attribute the paucity of dietary diversity and insufficient nutrition among WRA to the monotony of their meals, their low socioeconomic position, and a lack of nutrition assistance services for mothers [5,6]. In low- and middle-income countries (LMICs), starchy staples make up a large portion of whole meals, resulting in poor dietary diversity across all age groups. Bangladeshi women’s diets are also deficient in diversity and micronutrients [5,6]. A total of 54% of Bangladesh’s WRA eat a diet of basic grains and inadequate animal source items, such as fish, meat, eggs, and dairy [7]. As a result, the WRA may not consume enough micronutrients, putting themselves and their offspring at risk of deficiency and associated adverse health implications [8]. Despite the fact that supplementation can effectively reduce micronutrient deficiencies, program coverage is often low, and other techniques, such as biological fortification and other food-based interventions, require sufficient quantitative data prior to design and implementation.

Diversified diets may reduce nutritional deficits. Dietary diversity (DD) is a proxy for nutritional sufficiency because it shows how many different foods are eaten across and within food groups over a certain time period [9]. Macro-level WRA dietary quality assessment requires the FAO’s 10-food-group Minimum Dietary Diversity for Women (MDD-W) [10]. This strategy predicts women who eat five or more food types to meet their micronutrient needs more often than those who eat fewer. Individually, the MDD-W indicates nutritional adequacy by identifying food quality and micronutrient sufficiency [6,11]. The MDD-W separates women into high and low micronutrient sufficiency based on non-pregnant and non-lactating WRA dietary data. MDD-W statistics can help national, international, and non-governmental organizations support nutrition-sensitive policies and projects that diversify women’s diets.

Bangladesh instituted a countrywide lockdown in March 2020 after the WHO declared COVID-19 a pandemic. People and non-essential products and services were restricted from movement. Most people’s livelihoods were diminished [12,13]. The shutting of outdoor markets and small food businesses compounded the issue. Public transit closures impacted the food supply chain. Rapid food shortages made people more susceptible to acute food insecurity [14]. Evidence from South Asia, especially Bangladesh, indicates that throughout times of food crisis, intrahousehold food allocation is less beneficial to women, who sacrifice quantity and diversity of food to feed their families [15,16]. According to a paper that examined the indirect impacts of the COVID-19 pandemic on mother and child mortality, it was forecast that women in low- and middle-income nations will have a more difficult time obtaining healthful foods such as fruits and vegetables [17]. A recent review found that during the COVID-19 pandemic, women and people from poor socioeconomic backgrounds are likely to be more at risk of food insecurity [18]. However, few studies have quantified COVID-19’s influence on nutritionally disadvantaged Bangladeshi women’s food and nutrient consumption. Hence, the purpose of this study is to determine the state of women’s diet diversity using the MDD-W indicator and micronutrient adequacy during the pandemic-induced economic lockdown period. The study also dove into the possible determining factors of MDD-W in light of the COVID-19 crisis period.

## 2. Materials and Methods

### 2.1. Study Designs and Study Population

This survey was a cross-sectional study conducted in Chanpara Punarbasnkendra of Kayet Para Union within Rupganj, Narayanganj, Bangladesh. The study area is about 10 km northeast of Dhaka Zero Point, comprises about 5000 households, and is highly populated, like an urban slum. Though the study area is on a rural site, the facilities and other characteristics are like those in urban settlements. To better understand the study location and the areas where samples were collected, a Google map image (Supplementary Figure S1) was used to create a visual representation of the study site. For sample size calculation, we immediately assumed that 50% of the reproductive women might not meet the Minimum Dietary Diversity due to COVID-19. With a precision of 6.7% and a confidence interval of 95%, the minimum sample size required was 214. We surveyed 217 households, having reproductive-age women (age 15–49 years) and also having under 5 children, as discussed, in the previous study [13]. Pregnant and lactating women were excluded from this study. The average age of the study participants was 24.64 years (range: 16–36 years). An impact analysis was conducted during the middle of May to mid-June 2020, the initial days of the COVID-19 wave. A structured questionnaire was used to collect information on socio-demographic factors, including age, education, occupation, monthly family income before COVID-19 and income during the pandemic, and family members.

### 2.2. The Measure of Nutrient Consumption and Dietary Diversity

Dietary intake was collected using two consecutive 24 h diet recall methods. Two well-trained nutritionists collected the dietary data. Both of the enumerators are skilled in dietary assessment methods and all the measuring utensils were standardized before the final data collection. The plates, cups, and spoons were exhibited to achieve the closest feasible approximation of the amount of food eaten. The estimated cooked food portion was converted to equivalent raw food using the appropriate yield factor as reported in the “Food Composition Table for Bangladesh” [19]. The food consumption data of these selected individuals were evaluated using the “Food Composition Table for Bangladesh (FCTB-2013)” to calculate the nutrient content of the diet [19].

To measure the Minimal Dietary Diversity Score for Women (MDD-W), 24 h food recall was used to record the food and beverage consumed in the last 24 h by the individual. As recommended for MDD-W computation [10], all food products reported to be ingested during the first 24 h recall were divided into ten food groups according to the 2016 FAO guideline. The cumulative dietary variety score was based on whether the person ate the food group. Minimum dietary variety requires consuming at least 5 of the 10 food categories, and higher scores indicate more diversity.

### 2.3. Measurement of Nutrient Adequacy

The nutrient adequacy ratio (NAR) was determined for 11 micronutrients to predict adequate intake of calcium, magnesium, iron, zinc, thiamin, riboflavin, pyridoxine, folate, vitamin C, vitamin A, and vitamin D. The NAR value for a specific nutrient is the ratio of a respondent’s current nutrient intake to the Estimated Average Requirement (EAR) for the matching age group. The EAR values for the above-mentioned nutrients were obtained from ICMR-NIN [20]. The total of all NARs was divided by the number of nutrients evaluated (*n* = 11) to calculate the mean adequacy ratio (MAR). NAR values were trimmed at 1 so that a nutrient with a higher NAR might not counterbalance one with a lower NAR. To establish a comparison with prior multi-country investigations, an adequacy ratio of 0.6 was utilized as a cut-off point for nutritional adequacy [6,21].

### 2.4. Other Measurements

The Household Food Insecurity Access Scale (HFIAS) guideline version 3 was used to measure household food insecurity (HFI) [22]. The HFIAS is a continuous measure of the extent of food insecurity mainly associated with household access in the past 4 weeks. The HFIAS questionnaire consists of nine questions divided into three domains of food insecurity: (1) concern and uncertainty about the family’s food supply; (2) a change in diet quality; and (3) an insufficient amount of food consumed. The nine ‘frequency-of-occurrence’ questions were asked as a follow-up to each phenomenon question to examine how often the situation takes place. Each reply was then scored in a range of 0–3, with 0 denoting ‘no occurrence’, 1 denoting ‘rarely’, 2 denoting ‘sometimes’, and 3 denoting ‘often’. The total frequency of occurrence over the previous 30 days was calculated, and the household scores ranged from 0 to 27. HFI was classified into four groups based on guidelines: food secure (HFIAS = 0–1), mildly food insecure (HFIAS = 2–7), moderately food insecure (HFIAS = 8–11), and severely food insecure (HFIAS > 11) [22,23]. These four categories were further integrated in this study into a binary variable equaling 1 if moderately or severely food insecure and 0 otherwise.

Five food-related coping strategies were used to determine the reduced Coping Strategy Index (rCSI). The five food-related coping strategies are as follows: unable to eat preferred food, trying to borrow food or any kind of help, consuming a smaller meal, restricting adult consumption so that young children can eat, and skipping meals over the previous 7 days. A prior paper has a comprehensive computation of the CSI [24]. A higher score implies that a household has used more coping strategies. The overall rCSI score in this study is divided into three categories: no or poor coping (CSI = 0–3), medium (CSI = 4–9), and high coping (CSI = ≥10).

In accordance with standard procedure, anthropometric measurements were obtained from all participants by trained interviewers. A portable electronic scale was used to measure weight to the nearest 0.1 kg after the subjects removed their shoes and heavy clothing. The balances were frequently checked with the use of standard weights. Height was measured with a locally prepared portable height scale with an accuracy of 0.1 cm. Women’s Body Mass Index (BMI) was calculated as weight in kilograms/height in meter square.

### 2.5. Statistical Analysis

The statistical package SPSS 21 software for WINDOWS (SPSS Inc., Chicago, IL, USA) was used to conduct statistical analyses. Means and standard deviations were used to offer descriptive analysis (SD). Depending on the goal of the study, several potential contributing factors (such as employment, education level, BMI, and household food insecurity) were divided into two or more groups. We dichotomized households based on their monthly income: low (<8000 TK) and high (≥8000 TK). Those cut-off values were established as they represented the median value of the corresponding variable in our sample. To determine the association between risk factors and Minimum Dietary Diversity for Women, a bivariate analysis was performed using cross-table and chi-square testing. The major predictive factors for Minimum Dietary Diversity for Women were investigated using logistic regression analysis. The study’s dependent variable was MDD-W, which is classified as low and high dietary diversity. Women who consumed less than five food groups were in the non-diverse category, while those who consumed five or more food groups were categorized into the diverse group. The multivariate analysis included predictor variables such as total income of households, size of family, occupation and education level of women, age and BMI of women, state of food insecurity at household level, pandemic-related Coping Strategy Index, and mean micronutrient adequacy ratio. Descriptive statistics were used to present general characteristics of study subjects and the degree of association (unadjusted) between minimal dietary diversity and other factors was assessed using simple logistic regression. The final logistic regression model included all variables with *p*-values less than 0.25. The estimates of the strengths of associations were exhibited by the adjusted odds ratio (AOR) with a 95% confidence interval (CI). A *p* value of <0.05 was considered to be statistically significant.

### 2.6. Ethical Consideration

This study was performed according to the guidelines suggested by the Declaration of Helsinki, and the study protocol was reviewed and approved by the Research Ethics Committee-Faculty of Allied Health Sciences, Daffodil International University (Ref. No.: FAHSREC/DIU/2021/1008(1), Date: 2 May 2020).

After explaining the purpose of the survey to the participants, those who were willing to take part in the study gave informed consent, before the interview. Individuals under the age of 18 obtained consent from their parents or husbands.

## 3. Results

Table 1 describes the socio-demographic, household food insecurity, dietary diversity, and coping strategy information. About 65% of the women have a secondary level of education. The main occupation of the study subjects was garment worker (21.7%), and the remaining about 76.9% were housewives (Table 1). Most of the households (58.1%) had an income between 5001–10,000 BDT/month. The mean BMI of the study subjects was 23.58 ± 3.28 (25th percentile to 75th percentile of BMI was 21.52–25.32). About 24.4% of the people in the study were overweight (BMI 25.00–29.99) and 4.1% were obese (BMI ≥ 30). About two-thirds (59.9%) of the study subjects did not meet the MDD threshold, as they consumed foods from fewer than 5 food groups out of 10 food groups (Table 1). Throughout the study period, 6.9% of households were classified as food secure, 32.3% as slightly food insecure, 18.4% as moderately food insecure, and 42.4% as severely food insecure (Table 1). According to the CSI score, households used a couple of coping techniques to mitigate the impact of food shortages, with 47% using high coping strategies (Table 1).

Table 2 illustrates the Dietary Diversity Score (DDS) according to the socioeconomic factors and the nutritional status of the study subjects. The overall DDS of the reproductive-aged women was 4.34 ± 0.91, and the score was significantly high in the diverse group of women (Table 1 and Table 2). The descriptive analysis revealed that the number of study subjects differed significantly by study variable in the diverse and non-diverse diet groups (Table 2). Women’s education and nutritional status (BMI), family income, household food security, pandemic response strategy, and family size were all found to be significant factors in meeting the MDD of study participants (Table 2). Around one-fifth of primary/informal educated women, low-income women (8000 BDT/month), and women from low-coping-index-score families can reach the MDD (19.5%, 19.4%, and 18.6%, respectively) (Table 2). Malnourished women (underweight/overweight) did not fulfill the MDD in 71.2% of cases, whereas healthy women reached it in 54.2% of cases (BMI: 18.5–24.99) (Table 2). A higher percentage of women in moderate/severe food-insecure households (78%) were in the non-diverse diet group when compared to the food-secure/mildly food-insecure families (31.8%) (Table 2).

**Figure 1:**
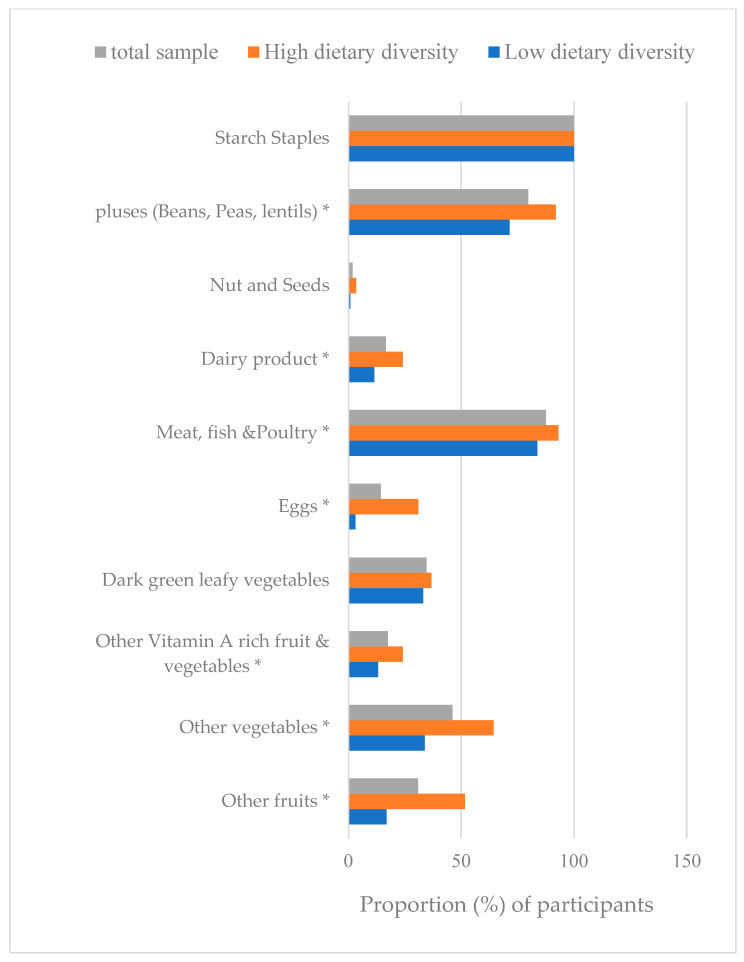
Proportion (%) of participants consuming each food group. Overall, diverse group consuming ≥ 5 food groups and non-diverse consuming < 5 food groups; * *p* < 0.05 significant by χ^2^ test within high- and low-diversify groups. MDD-W, Minimum Dietary Diversity for Women.

Figure 1 demonstrates the proportion of the reproductive women who attained the Minimum Dietary Diversity according to 10 food groups. Starchy, pulses, and flesh foods were the very most common food groups consumed by study subjects in both women of diverse (consuming ≥ five food groups) and non-diverse (consuming < five food groups) classes (Figure 1). The overall least consumption was for dairy products (16.6%), eggs (14.3), and nuts and seeds (1.8%). A significantly higher number of women in diverse groups consumed flesh foods (93.1%), pulses (92%), other vegetables (64.4%), other fruits (51.7%), eggs (31%), vitamin-A-rich fruits and vegetables (24.1%), and dairy products (24.1%) compared to females with non-diverse subgroups (83.8%, 71.5%, 33.8%, 16.9%, 3.1%, 13.1%, and 11.5%, respectively) (Figure 1). Supplementary Figure S2 outlines the most- and least-ingested foods.

Table 3 compares the nutrient consumption levels of study subjects with a diverse (DDS ≥ 5) versus a non-diverse diet (DDS < 5). The average energy and protein consumption of the participants were (mean ± SD) 1475.1 ± 191.3 kcal and 46.3 ± 9.9 gm, respectively. Both protein and energy consumption were significantly higher in the diverse diet group compared to the non-diverse diet group (Table 3). Except for vitamin C, vitamin A, and vitamin D, all nutrients evaluated (macro- and micronutrients) in the diversified diet group had significantly higher consumption than the non-diverse diet group (Table 3). In general, women who achieved Minimum Dietary Diversity consumed more macronutrients and micronutrients than those who did not.

Out of the 11 micronutrients evaluated, magnesium, zinc, and vitamin C showed adequacy ratios higher than 60% of EAR (NAR > 0.6) in the overall study population (Table 4). The overall NARs of iron, vitamin B1, and vitamin A were (mean ± SD) 0.534 ± 0.132, 0.594 ± 0.187, and 0.530 ± 0.380, respectively, which showed an adequacy ratio just above 50% of the EAR (Table 4). Vitamin D was the most deficient micronutrient of the study subjects, with an overall NAR of 0.073 ± 0.138. In the overall reproductive women, the mean adequacy ratio (MAR) for all micronutrients was 0.468 ± 0.096. The NAR values for all nutrients were higher in the diverse diet subgroup compared to the non-diverse diet group. As a result, the MAR values were significantly higher in the diverse diet group (Table 4). The NARs of magnesium, iron, zinc, vitamin B1, folate, vitamin C, and vitamin A were just above 50% of the EAR for the diverse diet group, whereas this was only for magnesium, zinc, vitamin B1, and vitamin C in the non-diverse diet group (Table 4). Pearson correlation coefficients were calculated for every NAR micronutrient with the DDS for the entire group of reproductive women. Except for vitamins C and D, all the NAR nutrients correlated positively and significantly with the DDS (Table 4).

Table 5 shows the things that both univariate and multivariate regression models found to affect the Minimum Dietary Diversity. In the simple (univariate) binary logistic regression analysis, family size, family income, women’s education level and BMI, household food insecurity status, Coping Strategy Index during the pandemic, and mean micronutrient adequacy ratio were all linked to women’s Minimum Dietary Diversity (Table 5). In the multivariate logistic regression analysis (Table 5), household size, women’s education, coping strategy, and MAR were found to be significant predictors of women’s Minimum Dietary Diversity. Women with a lower level of education (primary/informal) were 3.56 times more likely (CI: 1.53–8.306) to have low dietary diversity than those with a higher level of education (secondary and higher). Coping technique was found to be another determinant of dietary diversity. Women in households with a high coping strategy were 4.42 times more likely to have low dietary diversity than those in no- or low-coping-strategy households, while women in households with a medium coping strategy were 3.014 times more likely to have low dietary diversity than those in households with no or low coping strategies. Family size and dietary diversity were inversely associated.

## 4. Discussion

This study aimed to understand dietary diversity and nutrient adequacy, as well as their determining factors, among the WRA in Bangladesh during the COVID-19 pandemic. The analysis showed that the majority of respondents scored lower for dietary diversity than the 5-point cut-off proposed by the FAO [10]. Most of these women belonged to households of informal workers and were experiencing high levels of food insecurity. There are just a few studies that employed MDD-W to evaluate the food diversity of WRA in Bangladesh. As per the literature, no study reported Minimum Dietary Diversity for Women (MMD-W) to measure food consumption and diet variation during the COVID-19 period in Bangladesh [18].

Our results show that 59.9% of WRA, which is a small rise from previous data, had an insufficiently diversified diet. In a population census conducted before the pandemic [25], 55% of Bangladeshi women said they did not eat enough different foods, and the number was higher for rural women. Prior to the start of the COVID-19 lockdown, a second survey of teenage females in Bangladesh indicated that 55.4% of them had an insufficiently diverse diet [26]. On the other hand, during the pandemic lockdown, women in rural southern Bangladesh ate more of a variety of food categories than the current study sample [27].

Women who did not eat a lot of different foods ate a lot less meat, eggs, pulses, dairy, and other fruits and vegetables than other women. In addition, none of the women in the study group ate enough dairy, eggs, nuts, or seeds. On the other hand, they did eat enough starchy foods. Starchy foods are more resourceful in terms of delivering family meals at a lesser cost than protein sources and vegetables, which are more expensive and difficult to procure for the low-income population. Nguyen et al. [6] found that women in Bangladesh consumed 75% of their calories from starchy staples, with only a small amount from other dietary categories. Another recent Bangladeshi study also indicated that females had less food diversity and ate more cereals compared to males, suggesting that this trend is more rooted than previously thought [28]. In these studies, increased food prices and poor incomes were regularly highlighted as hurdles to augmenting the primary diet with healthy foods for impoverished women in developing nations, such as Bangladesh. Few studies have quantified the impact of COVID-19 on food consumption and dietary quality. One study found that during the pandemic, food consumption dropped dramatically for 15% of rural households and 24% of urban households in Bangladesh that were previously able to consume three meals every day [29]. However, an examination of the change in dietary patterns among rural southern Bangladeshi women showed that food groups, especially fruit, milk, and dairy, rose at follow-up from baseline over the first year of COVID-19, and the same was true of food-producing households in rural China [30]. Therefore, rural residents consumed a better-quality diet, as they might well be able to eat their own produce during food shortages, unlike urban dwellers.

Most of the women had MDD-W scores of less than five, which shows that micronutrient deficiencies are likely in this particular community. In the current study, dietary variety was strongly linked with the MAR. Although overall nutrient consumption was considerably higher in the diversified diet group, the majority of women’s intake seemed insufficient. The high dependence on rice as a primary source of energy in the diets of impoverished Bangladeshi women and children is well known, and it has been linked to poor dietary diversity and drastically insufficient intakes of numerous micronutrients [8]. No nutrient in the diverse or non-diverse groups had NAR values greater than 50%, with the exception of magnesium, zinc, thiamine, and vitamin C. Riboflavin, calcium, and vitamin D intake were not adequate in the study group, regardless of diet diversification status. The study of WRA in Latin American countries showed a shortfall of vitamin D, which is identical to the current findings [31]. The inadequate calcium and riboflavin dietary intakes may be explained by women’s poor dairy consumption, as reflected in the present study as well as in data from research in rural Bangladesh [8,26]. The present study demonstrated inadequate NARs for vitamin A, pyridoxine, folate, and iron among women irrespective of their dietary diversity situation. An analysis of the 2018 Bangladesh Integrated Household Survey (BIHS) panel data on food consumption revealed that despite the addition of legumes, eggs, milk, and dairy products to people’s diets, the quantity consumed accounted for just about a third of the amount, and meat intake remains less than half of the requirement, although rice intake exceeds the necessary level [32]. This again potentially indicates that, while many people may have consumed meals from those categories, the quantity ingested was most likely insufficient, as previously noted by Arsenault et al. [8]. Interestingly, the estimated values of calories and protein consumption from the sample of the present study appear to be lower than the reported values for women aged 19–40 in the 2018 Bangladesh Integrated Household Survey (BIHS) [32]. This suggests that the COVID-19 pandemic affected the WRA’s total food intake in Bangladesh, increasing their risk of nutritional deficiency, irrespective of the number of food groups consumed.

A very small percentage of the target population belonged to food-secure households. Those with limited dietary diversity had high levels of food insecurity and used coping techniques, with the latter emerging as a key driver of MDD-W. During times of food insecurity, women are more likely to use coping techniques that expose them to dietary compromises, such as poor consumption of macro- and micronutrients, decreased intake of fruits and vegetables, and a lack of diet diversity. Studies conducted in several contexts, including Burkina Faso, Mali, Bangladesh, and Ecuador, discovered that extremely food-insecure families had a lower risk of obtaining MDD-W [33,34,35]. Similarly, the majority of those surveyed in the current study reported employing food-based coping techniques in the face of severe food insecurity. COVID-19’s effects on food systems were projected to include severe food shortages and price increases in both rural and urban regions, leading to decreased availability of food and alterations in consumer preferences toward less healthy foods, thus affecting dietary diversity potentially for women [36]. During the first phase of the lockdown, the majority of the population employed food-based coping strategies, the most common of which was eating less-preferred or expensive foods, followed by reducing the size of meals and skipping meals [13]. According to studies conducted in Sub-Saharan Africa, lowering dietary variety, as well as modifying the quality and amount of foods ingested owing to the COVID-19 lockdown, was a prevalent approach for households to manage rising basic food costs [37]. In Pakistan, study findings revealed that COVID-19 had a greater impact on the diet diversity of female households than male households, resulting in the lower intake of specific nutrients and lower overall consumption of perishable and non-perishable food commodities, such as meat and meat products, in the former group [38,39].

Many people and organizations in the global food chain have been hurt by the pandemic in different ways. The COVID-19 scenario may have worsened food insecurity and nutrition in the informal sector, which made up the majority of our study group, due to job losses, income drops, and rising food prices [12,14]. Since COVID-19 began, people have been eating less nutrient-dense and expensive sources of calories, such as legumes, nuts, and animal-source foods, relative to nutrient-poor and cheaper ones (staples) [40,41]. Diets rich in nutrients and variety cost more than diets dominated by grains and starchy staples. High-quality, perishable goods are more susceptible to malfunctions in emergencies [42,43]. The triple burden of malnutrition (undernutrition, overweight and obesity, and micronutrient deficiencies) in low- and middle-income countries (LMICs) amidst fast urbanization is caused by cheaper calorie consumption and dietary diversity loss.

Another important aspect influencing women’s dietary variety in the present study is their education. Earlier research has shown that women’s education and nutrition quality are inextricably linked [26,44,45]. Women with greater educational levels are more likely to possess greater nutritional awareness and be wealthier, making them less vulnerable to inadequate dietary diversity [45]. It has also been shown that education can help women make more autonomous decisions and have better access to household resources that are vital for their nutritional condition [46]. Lower education has the most impact on dietary patterns among younger females, as it reduces their decision-making ability and control over food choices within their homes, contributing to their poor diet quality [26]. Multiple studies have demonstrated that during the COVID-19 pandemic, higher educational achievement had a protective effect against the diminishing of dietary diversity at the household level [47,48]. Education thus helps improve family food supply by expanding employment options, increasing working efficiency, providing access to health and nutrition knowledge, raising income, and diversifying, even during crisis periods, and for those who are vulnerable, as evidenced in the present study. A few limitations of our study must be recognized. Due to the COVID-19 pandemic, some problems arose for both interviewers and respondents. A 24 h food recall was used to determine how much food was eaten, and the accuracy of the data depends on how well the subjects or respondents remember. The sample size of the study was small and only included a small portion of people in Bangladesh. The findings of the research offered merely a glimpse into the dietary diversity and nutrient adequacy of the reproductive women in selected locations in Bangladesh, so establishing broad conclusions is difficult.

## 5. Conclusions

This research contributes to the evidence for the existing vulnerability of women to economic shocks, which majorly affect their diet quality. Dietary diversity was found to be one of the easily affected variables during public health emergencies, such as the COVID-19 pandemic, which has greatly impacted nutrient intake. Another notable finding is that the attainment of minimal diversity was not sufficient to achieve acceptable nutrient adequacy for women, pertaining to their low-quantity intake. The findings therefore highlight the urgent need for government preparedness and actions for future shocks and pandemic-related sanctions in order to ensure adequate nutritional quality even during emergencies. They also showcase the need for women’s empowerment through education and the establishment of effective intrahousehold food distribution to maintain dietary diversity.

## Figures and Tables

**Table 1:** Socio-demographic, nutritional, and food security status of the study subjects.

Variables	Frequency	Percentage
Total women	217	100
Distribution by age:		
16–19 year	35	16.1
20–25 year	83	38.2
26–30 year	78	36.0
31–36 year	21	9.7
Occupation of women:		
Garment workers	47	21.7
Housewife	167	76.9
Private job worker and others	3	1.4
Educational status:		
Illiterate/informal education	5	2.3
Primary school	72	33.2
Secondary school	128	59.0
Higher secondary or above	12	5.5
Family income during COVID-19 lockdown:		
4000–5000	45	20.9
>5000–10,000	125	58.1
>10,000–15,000	33	15.4
>15,000–20,000	12	5.6
Nutritional status (BMI) (WHO criteria):		
Chronic energy deficiency (BMI < 18.5)	11	5.1
Normal (BMI 18.5–24.99)	144	66.4
Overweight (BMI 25–29.99)	53	24.4
Obese (BMI ≥ 30)	9	4.1
Dietary diversity:		
Low MDDS (0–4)	130	59.9
Acceptable MDDS (5–10)	87	40.1
Overall MDDS (mean ± SD)	4.34 ± 0.91	
Household food insecurity access prevalence:		
Food secure (Score 0–1)	15	6.9
Mildly food insecure (score 2–7)	70	32.3
Moderately food insecure (score 8–11)	40	18.4
Severely food insecure (score > 11)	92	42.4
CSI score		
No/low coping (0–3)	65	30.0
Medium coping (4–9)	50	23.0
High coping (>9)	102	47.0

BMI, Body Mass Index; MDDS, Minimum Dietary Diversity Score; CSI, Coping Strategy Index.

**Table 2:** Factor associated for meeting Minimum Dietary Diversity.

Factor	Non-Diverse Group (DDS < 5)	Diverse Group (DDS ≥ 5)	λ^2^ Test, *p* Value
DDS (mean ± SD)	3.708 ± 0.49	5.288 ± 0.48	<0.001 (*t* test)
Education			
Primary/informal	62 (80.5%)	15 (19.5%)	<0.001
Secondary to higher	68 (48.6%)	72 (51.4%)	
Age			
Less than 25 years	71 (65.1%)	38 (34.9%)	0.114
25 and above	59 (54.6%)	49 (45.4%)	
Occupation			
Unemployed/housewife	103 (61.3%)	65 (38.7%)	0.435
Employed	27 (55.1%)	22 (44.9%)	
Family Income			
<8000 BDT/ month	83 (80.6%)	20 (19.4%)	<0.001
≥8000 BDT/month	45 (40.2%)	67 (59.8%)	
BMI			
Normal	78 (54.2%)	66 (45.8%)	0.015
Underweight/overweight	52 (71.2%)	21 (28.8%)	
HFISA			
Moderately/severely food insecure	103 (78%)	29 (22%)	<0.001
Mildly insecure to food secure	27 (31.8%)	58 (68.2%)	
Coping index			
Low coping	18 (27.7%)	47 (72.3%)	<0.001
Moderate coping	29 (58.0%)	21 (42.0%)	
High coping	83 (81.4%)	19 (18.6%)	
Family size			
≥4	28 (44.8%)	32 (55.2)	0.006
<4	102 (65.0%)	55 (35.0%)	

DDS, Dietary Diversity Score; BMI, Body Mass Index; HFISA, Household Food Insecurity Access Scale.

**Table 3:** Consumption of energy and nutrients of study subjects in diverse and non-diverse diet groups.

Variables	Overall		Non-Diverse Group (DDS < 5)		Diverse Group (DDS ≥ 5)		*p* Value
	Mean	SD	Mean	SD	Mean	SD	
Energy (Kcal)	1475.1	191.3	1418.2	165.0	1560.1	196.1	<0.001
Protein (g)	46.3	9.9	42.64	8.24	51.68	9.97	<0.001
Carbohydrates (g)	256.8	32.9	250.2	30.37	265.5	34.47	<0.001
Fat and oil (g)	25.2	8.2	23.47	7.78	27.76	8.24	<0.001
Dietary fiber (g)	18.7	3.6	17.87	3.35	19.87	3.68	<0.001
Calcium (mg)	195.8	110.0	180.98	95.26	217.93	126.41	0.015
Magnesium (mg)	247.2	45.8	238.95	48.95	259.47	37.81	<0.001
Iron (mg)	8.1	1.9	7.65	1.67	8.87	2.02	<0.001
Zinc (mg)	6.8	1.1	6.44	0.93	7.24	1.20	<0.001
Copper (mg)	1.2	0.2	1.19	0.26	1.27	0.24	0.023
Vitamin B1 (mg)	0.7	0.2	0.663	0.25	0.74	0.27	0.045
Vitamin B2 (mg)	0.37	0.12	0.35	0.11	0.41	0.13	<0.001
Vitamin B6 (mg)	0.64	0.15	0.60	0.16	0.70	0.13	<0.001
Folate (µg)	90.3	32.2	86.83	35.0	95.41	26.85	<0.001
Vitamin C (mg)	54.7	43.1	50.27	40.71	61.45	45.9	0.061
Vitamin A (µg)	355.1	396.	315.61	369.2	414.23	428.54	0.072
Vitamin D (µg)	0.73	1.3	0.70	1.37	0.76	1.41	0.730

DDS, Dietary Diversity Score.

**Table 4:** Nutrient adequacy ratio (NAR) of specific nutrients in different groups and its correlation with Dietary Diversity Score.

Nutrients	Overall		Non-Diverse Group		Diverse Group		*p* **	Pearson Correlation *	
	Mean	SD	Mean	SD	Mean	SD	*p*	r	*p*
Calcium	0.243	0.136	0.224	0.118	0.271	0.156	0.013	0.171	0.012
Magnesium	0.785	0.119	0.756	0.120	0.828	0.105	<0.001	0.335	<0.001
Iron	0.534	0.132	0.499	0.116	0.586	0.138	<0.001	0.317	<0.001
Zinc	0.610	0.102	0.580	0.084	0.655	0.110	<0.001	0.454	<0.001
Vitamin B1	0.594	0.187	0.568	0.182	0.634	0.189	0.011	0.262	<0.001
Vitamin B2	0.230	0.078	0.214	0.073	0.253	0.080	<0.001	0.288	<0.001
Vitamin B6	0.396	0.100	0.369	0.099	0.435	0.087	<0.001	0.364	<0.001
Folate	0.489	0.169	0.467	0.182	0.522	0.143	0.019	0.173	0.011
Vitamin C	0.664	0.336	0.638	0.335	0.704	0.335	0.153	0.107	0.115
Vitamin A	0.530	0.380	0.491	0.384	0.589	0.369	0.062	0.144	0.034
Vitamin D	0.073	0.138	0.070	0.137	0.077	0.141	0.730	0.061	0.373
MAR	0.468	0.096	0.444	0.094	0.505	0.087	<0.001	0.365	<0.001

NAR: nutrient adequacy ratio. MAR: mean adequacy ratio. DDS < 5: non-diverse diet. DDS ≥ 5: diverse diet. SD: standard deviation. * Pearson correlation coefficients (r) were calculated between each NAR value and the DDS for the whole sample. ** Independent sample *t* test between diverse and non-diverse groups.

**Table 5:** Model of logistic regression for the prediction of Minimum Dietary Diversity.

	Univariate			Multivariate		
	OR	95% CI of ORs	Sig	AOR	95% CI of AORs	Sig
Family Size: family member ≤ 3	2.327	1.262–4.292	0.007	2.389	1.07–5.33	0.034
Family member > 3 (r)						
Monthly income during pandemic: <8000 (BDT)	6.179	3.333–11.455	<0.001	1.343	0.519–3.477	0.543
Income ≥ 8000 (r)						
Age of respondents: <25 years	1.552	0.898–2.680	0.115	1.242	0.608–2.535	0.553
Age ≥ 25 years (r)						
Occupation: Housewife/unemployed	1.291	0.679–2.456	0.436	Remove from here		
Garment/service worker (r)						
Education: Primary/informal	4.376	2.275–8.418	<0.001	3.567	1.53–8.306	0.003
Secondary/higher (r)						
BMI: Underweight/overweight	2.095	1.146–3.831	0.016	1.601	0.761–3.388	0.214
Normal (18.5–24.99) (r)						
HFI: Moderately to severely food insecure	7.63	4.125–14.113	<0.001	1.528	0.495–4.714	0.461
Secure to mildly food insecure (r)						
HH Coping Strategy Index (CSI):			<0.001			0.074
Medium (4–9)	3.606	1.651–7.877	<0.001	3.014	1.09–8.36	0.034
High (>9)	11.41	5.457–23.483	<0.001	4.42	1.05–18.53	0.042
No/low (0–3) (r)						
Micronutrient Adequacy Ratio	0.001	0.000–0.017	<0.001	0.002	0.000–0.118	0.002
Constant				2.052		0.531

BMI, Body Mass Index; HFI, household food insecurity; HH, household; OR, odds ratio; AOR, adjusted odds ratio.

## Data Availability

All the data for this study are available in the manuscript.

## Supplementary Materials

The following supporting information can be downloaded at: https://www.mdpi.com/article/10.3390/nu15143202/s1, Figure S1: Location of the studied area in Chanpara, Rupganj, Narayanganj (23°43′49.70″ N; 90°30′09.92″ E), shown using a red polygon. The background is the Google Earth image. Figure S2: The names of food that were consumed by the study subjects during the COVID-19 pandemic. From the list, the most frequently consumed foods were rice, lentil, ruti, potato, and tea, and least frequently consumed foods were beef, mola carplet, pomelo, cowpea, carrot, and brinjal.
