# Supplementary material for: Impact of COVID-19 on Micronutrient Adequacy and Dietary Diversity among Women of Reproductive Age from Selected Households in Bangladesh
Source: Nutrients. 2023 Jul 19;15(14):3202. doi: 10.3390/nu15143202 (PMC10386131; doi:10.3390/nu15143202)
Supplement: Supplementary file 1 [file nutrients-15-03202-s001.zip › Supplementary Fig_1.pdf]

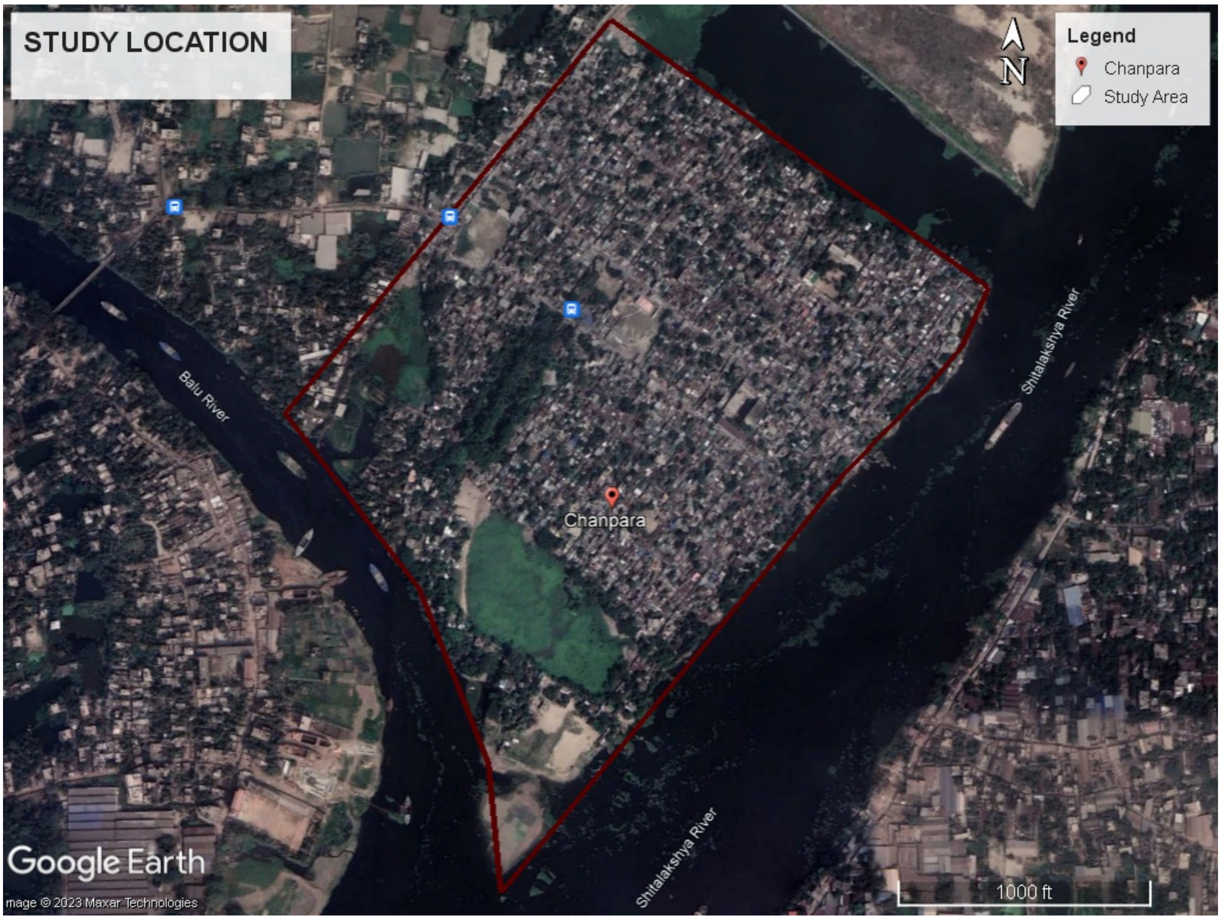

Supplementary Figure S1: Location of the studied area in Chanpara, Rupganj, Narayanganj (23°43′49.70″ N; 90°30′09.92″ E), showing using red polygon. The background is the Google Earth Image.
