# Supplementary material for: Impact of COVID-19 on Micronutrient Adequacy and Dietary Diversity among Women of Reproductive Age from Selected Households in Bangladesh
Source: Nutrients. 2023 Jul 19;15(14):3202. doi: 10.3390/nu15143202 (PMC10386131; doi:10.3390/nu15143202)
Supplement: Supplementary file 1 [file nutrients-15-03202-s001.zip › Supplementary Fig_2.pdf]

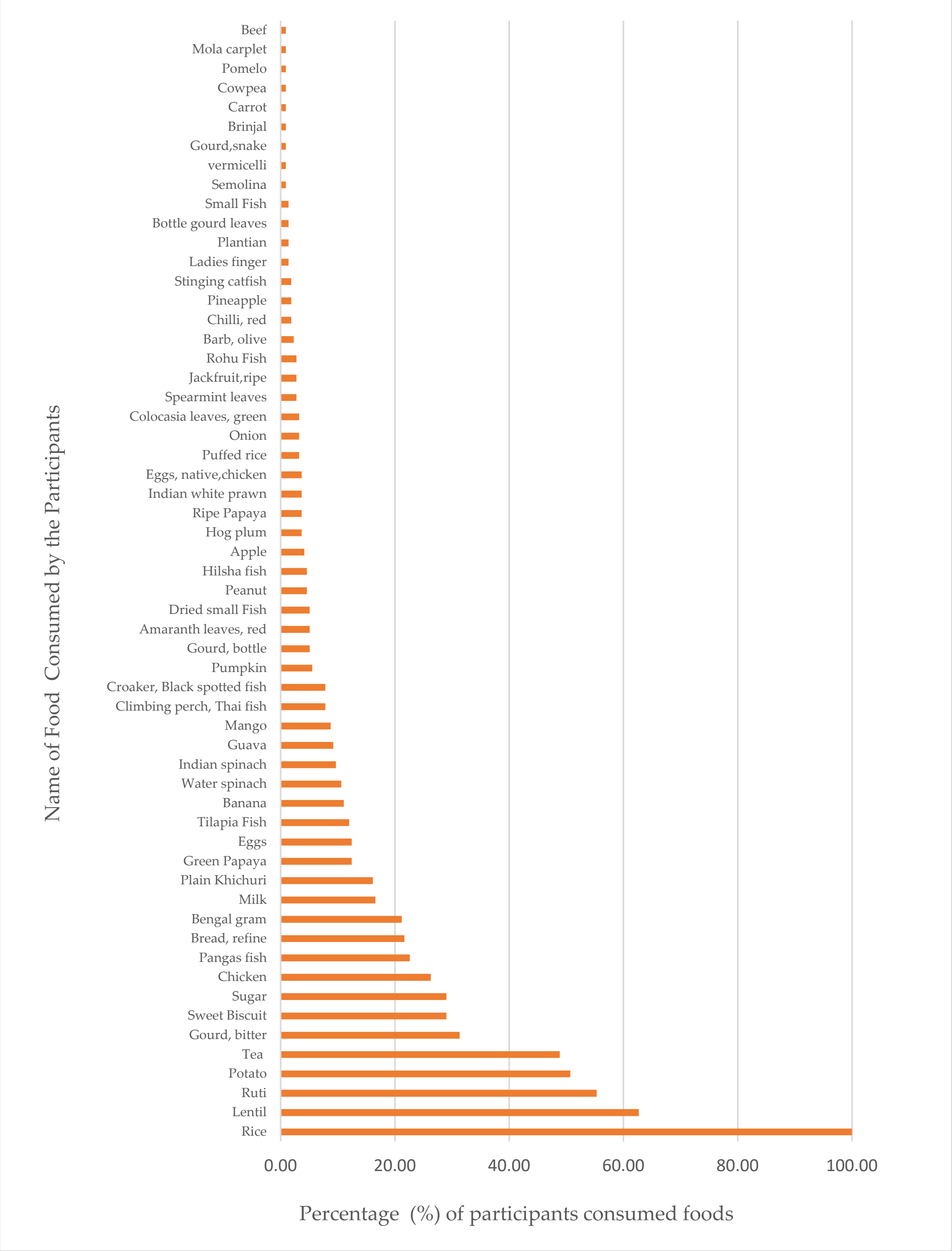

Supplementary Figure S2: The Name of Food that are consumed by the study subject during the COVID-19 pandemic. Form the list the most frequently consumed foods were rice, lentil, ruti, potato, tea etc; and least frequently consumed food were beef, mola carplet, pomelo,cowpea, carrot and brinjal.
